# Simulation of Hemorrhage Pathogenesis in Mice through Dual Stimulation with Dengue Envelope Protein Domain III-Coated Nanoparticles and Antiplatelet Antibody

**DOI:** 10.3390/ijms24119270

**Published:** 2023-05-25

**Authors:** Te-Sheng Lien, Der-Shan Sun, Wen-Sheng Wu, Hsin-Hou Chang

**Affiliations:** 1 Department of Molecular Biology and Human Genetics, Tzu-Chi University, Hualien 970, Taiwan; alan211@mail.tcu.edu.tw (T.-S.L.); dssun@mail.tcu.edu.tw (D.-S.S.); 2 Division of General Surgery, Department of Surgery, Hualien Tzu Chi Hospital, Buddhist Tzu Chi Medical Foundation, Hualien 970, Taiwan; wuws@gms.tcu.edu.tw

**Keywords:** dengue virus, dengue envelope protein domain III, dengue hemorrhage fever, hemorrhage, antiplatelet antibody, silica nanoparticles, inflammation, cytokines, anti-coagulants, two-hit model

## Abstract

Dengue hemorrhagic fever (DHF) is a severe form of dengue virus (DENV) infection that can lead to abnormal immune responses, endothelial vascular dysfunction, and hemorrhage pathogenesis. The virion-associated envelope protein domain III (EIII) is thought to play a role in the virulence of DENV by damaging endothelial cells. However, it is unclear whether EIII-coated nanoparticles simulating DENV virus particles could cause a more severe pathogenesis than soluble EIII alone. This study aimed to investigate whether EIII-coated silica nanoparticles (EIII-SNPs) could elicit greater cytotoxicity in endothelial cells and hemorrhage pathogenesis in mice compared to EIII or silica nanoparticles alone. The main methods included in vitro assays to assess cytotoxicity and in vivo experiments to examine hemorrhage pathogenesis in mice. EIII-SNPs induced greater endothelial cytotoxicity in vitro than EIII or silica nanoparticles alone. Two-hit combined treatment with EIII-SNPs and antiplatelet antibodies to simulate DHF hemorrhage pathogenesis during secondary DENV infections resulted in higher endothelial cytotoxicity than either treatment alone. In mouse experiments, two-hit combined treatment with EIII-SNPs and antiplatelet antibodies resulted in more severe hemorrhage pathogenesis compared to single treatments of EIII, EIII-SNPs, or antiplatelet antibodies alone. These findings suggest that EIII-coated nanoparticles are more cytotoxic than soluble EIII and could be used to develop a tentative dengue two-hit hemorrhage pathogenesis model in mice. Additionally, our results indicated that EIII-containing DENV particles could potentially exacerbate hemorrhage pathogenesis in DHF patients who have antiplatelet antibodies, highlighting the need for further research on the potential role of EIII in DHF pathogenesis.

## 1. Introduction

Dengue virus (DENV) infection can lead to self-limited dengue fever and live-threatening dengue hemorrhagic fever (DHF) [1,2,3]. Despite the detailed mechanism remains to be further investigated, secondary DENV infection increases risk of DHF. This suggests that anti-viral immunity may contribute to the lethal pathogenesis [1,2,3].

When a person is infected with dengue virus for the first time, the immune system produces antibodies that help fight the virus. However, if the same person is infected with a different strain of the virus during a secondary infection, the antibodies produced during the first infection may make the second infection worse [1,2,3]. During a secondary infection, the antibodies produced in response to the first infection can bind to the new virus but are not effective in neutralizing it. Instead, the production of host antigen cross-reactive autoantibodies may lead to an excessive immune response and increased production of cytokines. This can cause damage to blood vessels and bleeding, which could be potentially severe and life-threatening [4,5,6,7,8,9].

Nanotechnology has various applications in biomedical science research, including antimicrobial substances, drug delivery, imaging, tissue engineering, and biosensors [10,11,12,13,14,15,16]. Nanoscale virus-like particles can be engineered to mimic virus particles and stimulate the immune system to produce an immune response, without causing an actual infection. To achieve this, virus-like particles were designed to resemble the size, shape, and surface proteins of a virus particle. The surface of the nanoparticles could be modified to mimic the viral proteins that are recognized by the immune system, known as antigens. Upon exposure to cells or injection into the body, nanoparticles are perceived as foreign entities and can stimulate cellular or immune responses, resembling those elicited by a pathogen encounter [17,18,19,20,21,22,23,24]. This study will utilize virus protein-coated particles to investigate the pathogenic impact of the DENV envelope protein domain III (EIII) in a two-hit model. Previous studies indicate that autoantibodies produced by the DENV viral protein can have cytotoxic effects [6,8,9,25] and contain antiplatelet fractions [4,5,9]. Moreover, the combined administration of EIII and antiplatelet autoantibodies have been found to induce cell death and aggravate hemorrhagic pathogenesis in mice [7,26,27]. The objective of this study is to determine whether EIII-coated nanoparticles will have a similar or exacerbated impact by cross-linking EIII-binding molecules on the cell surface in an EIII plus antiplatelet antibody co-treated two-hit model. Results showed that combined treatments of EIII-coated virus-like nanoparticles and antiplatelet autoantibodies induced greater endothelial cell death in vitro and exacerbated hemorrhage pathogenesis in mice. The potential applications of the EIII-coated virus-like nanoparticles and the two-hit induced hemorrhage mouse model are also discussed.

## 2. Results

### 2.1. EIII-Coated Nanoparticles Displayed Enhanced Cytotoxicity against Endothelial Cells

**Figure 1:**
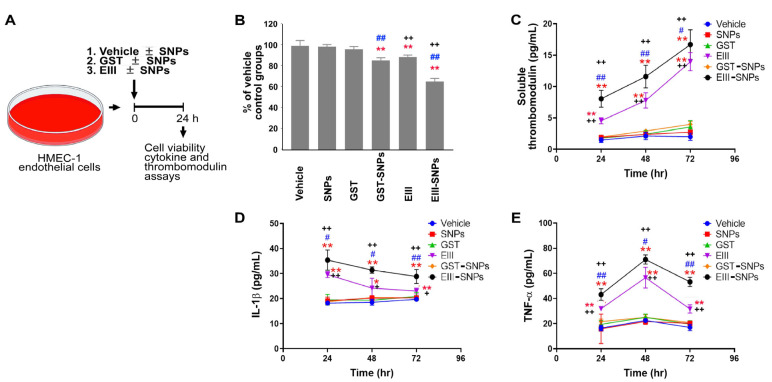
Endothelial HMEC-1 cell survival after GST and EIII treatments. (**A**) Experiment outline. (**B**) Cell viability, (**C**) soluble form thrombomodulin, (**D**) IL-1β, and (**E**) TNF-α of human endothelial HMEC-1 cells were analyzed to examine the cytotoxicity of GST, EIII, GST-SNPs, and EIII-SNPs treatments. Vehicle, GST, and EIII groups, N = 12; SNPs, GST-SNPs and EIII-SNPs groups, N = 6. * *p* < 0.05, ** *p* < 0.01 vs. vehicle groups; # *p* < 0.05, ## *p* < 0.01 vs. respective soluble protein (without SNP) groups; + *p* < 0.05, ++ *p* < 0.01 vs. respective GST-SNP groups. Data are presented as mean ± SD.

To assess whether EIII-coated nanoparticles exhibit increased cytotoxicity against human HMEC-1 endothelial cells, the cells were treated with recombinant GST (a control protein), EIII, nanoscale silica beads (50 nm; SNPs), GST-coated SNPs (GST-SNPs), and EIII-coated SNPs (EIII-SNPs). The cell viability data showed that EIII displayed higher cytotoxicity than GST treatments. Additionally, EIII-SNPs exhibited higher cytotoxicity than the GST-SNPs and treatments of EIII alone (Figure 1A, experiment outline; Figure 1B, surviving cell levels). The levels of the endothelial cell injury marker, soluble form thrombomodulin, and pro-inflammatory cytokines IL-1β and TNF-α were consistently elevated in the EIII-SNP groups compared to the control and EIII-treated groups (Figure 1C–E). This indicated that soluble form EIII indeed increased its cytotoxicity after being coated on SNP surfaces.

### 2.2. Treatments with EIII-SNPs Induced Exacerbated Thrombocytopenia in a Two-Hit Mouse Model

**Figure 2:**
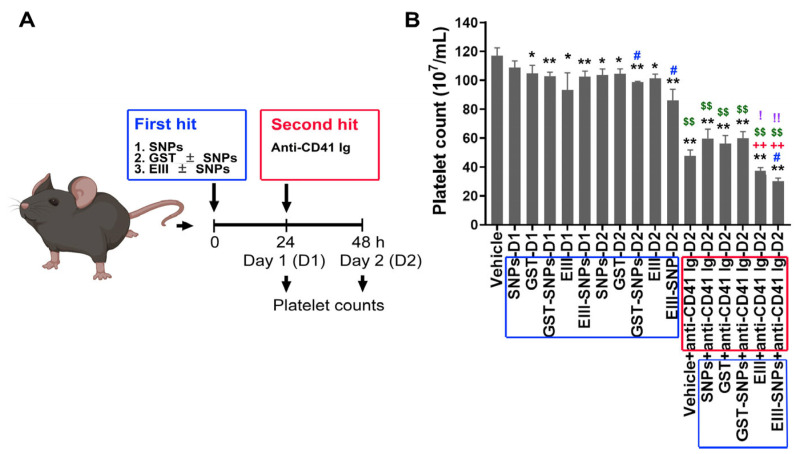
Platelet counts after GST and EIII treatments in C57BL/6J mice. (**A**) Experiment outline. (**B**) Platelet counts were analyzed to examine the severity of thrombocytopenia progression in mice groups without (vehicle), and with first hits (GST, EIII, GST-SNPs, and EIII-SNPs), and with two-hit challenges (first hits plus the second hit antiplatelet Ig treatments). D1: 24 h after first hit treatments; D2: 48 h after first hit treatments. Blue boxes: first-hit treatments; red boxes: second-hit treatments. N = 6. * *p* < 0.05, ** *p* < 0.01, vs. vehicle control groups; # *p* < 0.05, vs. respective soluble protein (without SNP) groups; ++ *p* < 0.01, vs. respective GST groups; $$ *p* < 0.01, vs. respective first-hit groups; ! *p* < 0.05, !! *p* < 0.01, vs. vehicle + anti-CD41 groups. Data are presented as mean ± SD.

Following previously described methods [6,27], a two-hit mouse model was employed. To investigate whether EIII-SNPs could induce a worsened thrombocytopenia phenotype in vivo, C57BL/6J mice were subjected to first-hit treatments of GST, EIII, SNPs, GST-SNPs, and EIII-SNPs for 24 h, and then followed by additional 24 h second-hit treatment of antiplatelet (anti-CD41) antibody. Analysis results revealed that two-hit EIII-SNPs plus anti-CD41 treatments indeed induced exacerbated thrombocytopenia in mice when compared to the other control groups (Figure 2A, experiment outline; Figure 2B, mouse platelet counts).

### 2.3. Treatments of EIII-SNPs Induced a Worsen Hemorrhage Pathogenesis in a Two-Hit Mouse Model

**Figure 3:**
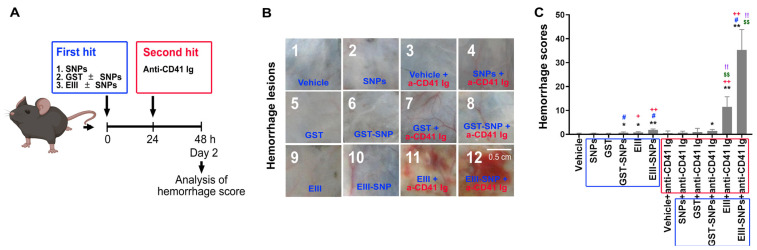
Hemorrhage manifestations after two-hit treatments of EIII plus antiplatelet Ig in mice. Following previously described methods [6,7] ((**A**), experiment outline), images of the hemorrhage lesions of mice were photographed (**B**), and the hemorrhage scores (**C**) were analyzed to examine the severity of hemorrhage pathogenic progression in mice groups without (vehicle), and with first hits (GST, EIII, GST-SNPs, EIII-SNPs), and with two-hit challenges (first hits plus the second hit antiplatelet Ig [anti-CD41 Ig: a-CD41 Ig] treatments). Blue boxes: first-hit treatments; red boxes: second-hit treatments. N = 4. * *p* < 0.05, ** *p* < 0.01, vs. vehicle control groups; # *p* < 0.05, vs. respective soluble protein (without SNP) groups; + *p* < 0.05, ++ *p* < 0.01, vs. respective GST groups; $$ *p* < 0.01, vs. respective first-hit groups; !! *p* < 0.01, vs. vehicle + anti-CD41 groups. Data are presented as mean ± SD. Scale bar: 0.5 cm.

Hemorrhage is a hallmark symptom in DHF [1] and could be reproduced in dengue two-hit mouse models [6,27]. To investigate whether EIII-SNPs could induce a worsened phenotype of hemorrhage pathogenesis in vivo, following previously described methods [6,27], C57BL/6J mice were subjected to treatments of GST, EIII, SNPs, GST-SNPs, and EIII-SNPs for 24 h as first-hits, and then followed by 24 h treatment of antiplatelet (anti-CD41) antibody as second-hit. Our data revealed that EIII-SNPs plus anti-CD41 two-hit treatments induced a worsened hemorrhage manifestation in mice (Figure 3A, experiment outline; Figure 3B, example hemorrhage images; Figure 3C, mouse hemorrhage score).

### 2.4. Treatments of EIII-SNPs Induced Exacerbated Inductions of Pro-Inflammatory Cytokines in a Two-Hit Mouse Model

**Figure 4:**
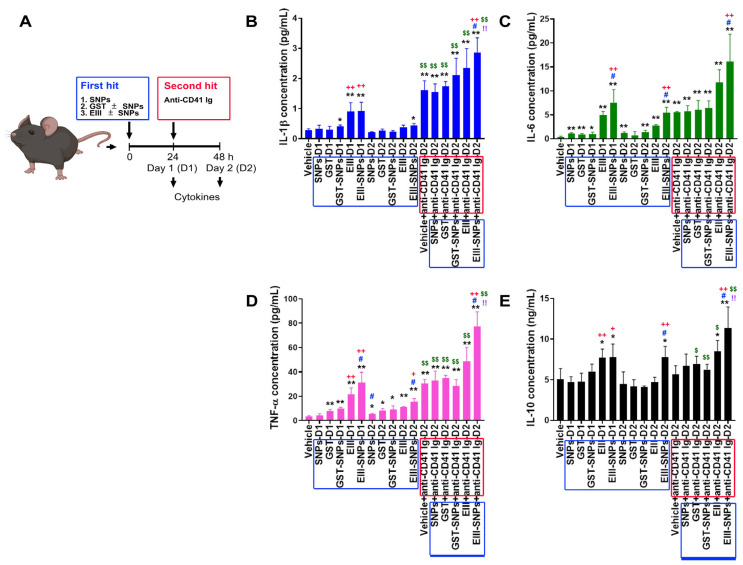
Circulating cytokine levels after two-hit treatments of EIII plus antiplatelet Ig in mice. (**A**) Experiment outline. (**B**–**E**) Enzyme-linked immunosorbent assay was performed to examine the expression of proinflammatory cytokines IL–1β (**B**), IL–6 (**C**), TNF–α (**D**), and anti-inflammatory cytokine IL–10 (**E**) in mice groups without (vehicle), and with first hits GST, EIII, GST-SNPs, EIII-SNPs, and first hits plus the second hit antiplatelet Ig treatments. D1: 24 h after first hit treatments; D2: 48 h after first hit treatments. Blue boxes: first-hit treatments; red boxes: second-hit treatments. N = 6. * *p* < 0.05, ** *p* < 0.01, vs. vehicle control groups; # *p* < 0.05, vs. respective soluble protein (without SNP) groups; + *p* < 0.05, ++ *p* < 0.01, vs. respective GST groups; $ *p* < 0.05, $$ *p* < 0.01, vs. respective first-hit groups; !! *p* < 0.01, vs. vehicle + anti-CD41 groups. TNF–α, tumor necrosis factor–α; IL–1β, interleukin–1β; IL–6, interleukin 6; IL–10, interleukin 10. Data are presented as mean ± SD.

Cytokine storms are critical for the development of DHF [28,29,30]. More specifically, pro-inflammatory cytokines TNF-α, IL-1β, IL-6, and anti-inflammatory cytokine IL-10 have been shown to be elicited in DHF patients, and in dengue two-hit mouse models [6,7,27,28,29,30]. Here, we would like to investigate whether EIII-SNPs could induce a worsened phenotype on the elicitation of these cytokines in vivo. Mice were subjected to 24 h first-hit treatments of GST, EIII, SNPs, GST-SNPs, and EIII-SNPs, and then followed by 24 h second-hit treatment of antiplatelet (anti-CD41) antibody. Our data revealed that EIII-SNPs plus anti-CD41 two-hit treatments induced exacerbated pro-inflammatory cytokine TNF-α, IL-1β, IL-6, and anti-inflammatory cytokine IL-10 inductions in mice (Figure 4A, TNF-α levels; Figure 4B, IL-1β levels; Figure 4C, IL-6 levels; Figure 4D, IL-10 levels).

### 2.5. Treatments of EIII-SNPs Induced Suppression of Anti-Coagulant Activated Protein C and Antithrombin III

**Figure 5:**
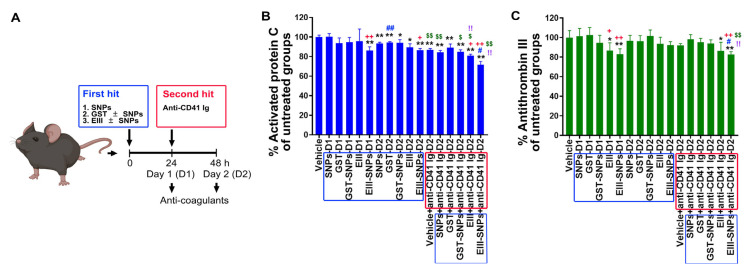
Circulating anti-coagulant activated protein C levels after two-hit treatments of EIII plus antiplatelet Ig in mice. (**A**) Experiment outline. (**B**,**C**) Activated protein C (**B**) and antithrombin III (**C**) analyses were performed to examine the expression levels of circulating aPC in mice groups without (vehicle), and with first hits GST, EIII, GST-SNPs, EIII-SNPs, and first hits plus the second hit antiplatelet Ig treatments. D1: 24 h after first hit treatments; D2: 48 h after first hit treatments. Blue boxes: first-hit treatments; red boxes: second-hit treatments. N = 6. * *p* < 0.05, ** *p* < 0.01, vs. vehicle control groups; # *p* < 0.05, ## *p* < 0.01, vs. respective soluble protein (without SNP) groups; + *p* < 0.05, ++ *p* < 0.01, vs. respective GST groups; $ *p* < 0.05, $$ *p* < 0.01, vs. respective first-hit groups; !! < 0.01, vs. vehicle + anti-CD41 groups. Data are presented as mean ± SD.

Hypercoagulation may induce disseminated intravascular coagulation (DIC) and then lead to secondary hemorrhage in DHF [31]. Hypercoagulation and DIC could be revealed by the manifestation of suppressed anti-coagulant factors [9,32]. Accordingly, here we would like to investigate whether EIII-SNPs could induce an exacerbated phenotype on the suppression of anti-coagulant factors in vivo. Experimental mice were subjected to first-hit treatments of GST, EIII, SNPs, GST-SNPs, and EIII-SNPs, and then 24 h second-hit treatment of antiplatelet (anti-CD41) antibody as described. We found that EIII-SNPs plus anti-CD41 two-hit treatments induced an exacerbated suppression of anti-coagulant-activated protein C and antithrombin III levels in mice (Figure 5). These results collectively suggested that, when compared to soluble EIII, EIII-SNP could be a more potent material for the development of dengue two-hit hemorrhage pathogenesis mouse model.

## 3. Discussion

This study demonstrates that EIII-SNPs (EIII-coated nanoparticles) have advantages over the soluble EIII to perform two-hit dengue hemorrhage mouse model because they induce enhanced cytotoxicity against endothelial cells and display higher cytotoxicity when compared to the GST-SNPs and respective treatments of EIII and SNPs alone. Additionally, EIII-SNPs induce exacerbated thrombocytopenia, worsen hemorrhage pathogenesis, and the induction of pro-inflammatory cytokines TNF-α, IL-1β, IL-6, and anti-inflammatory cytokine IL-10 in mice. Moreover, EIII-SNPs induce an exacerbated suppression of anti-coagulant activated protein C and antithrombin III levels in mice, indicating that they may be a more potent and virus particle-like material for the development of dengue two-hit hemorrhage pathogenesis mouse models than soluble EIII.

There are several examples in the literature that demonstrate the enhanced potency of cell-surface binding proteins coated on nanoparticles compared to their non-coated forms. One such example is a chimeric *Leishmania infantum* protein, which has been shown to induce a stronger and more sustained immune response when coated on poly(lactic-co-glycolic acid) nanoparticles [33]. Similarly, human papillomavirus protein coated on polyethyleneimine-functionalized nanoparticles demonstrated enhanced immunogenicity and improved protective efficacy compared to the non-coated protein [34]. These studies suggest that nanoparticle coating can enhance the potency of cell-surface binding proteins. Despite the fact that the underlying mechanism remains to be further elucidated, these factors may contribute to the increased potency of cell signaling induced by EIII protein when it is coated on nanoparticles. The increased surface area of the nanoparticles can also enhance the avidity of the binding protein, meaning that multiple binding events can occur simultaneously, resulting in a stronger interaction with the receptor [34,35,36]. In addition, it is possible that a receptor cross-linking effect is involved, where the binding protein on the nanoparticles can bring multiple receptors into close proximity, promoting the formation of receptor clusters and the initiation of downstream signaling pathways [37]. However, the exact mechanism of this phenomenon remains unclear and warrants further investigation.

**Figure 6:**
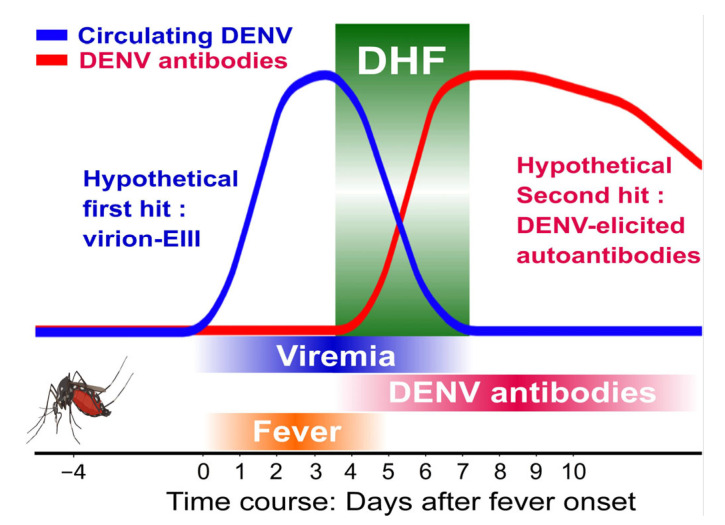
The illustration depicts the pathogenesis of DHF during secondary DENV infection, along with the timeline of mosquito bite (4 days prior to fever), circulating DENV increase (a hypothetical first hit: virion-associated EIII; blue labels) and DENV-elicited antibody (a hypothetical second hit: DENV-elicited autoantibodies; red labels) in DHF patients. The development of DHF coincides with the co-occurrence of viremia and DENV antibody production, suggesting a two-hit mechanism. Accordingly, here we hypothesized that DHF can only occur when both hits, namely viremia (surface-EIII containing nanoparticles as surrogates in this report) and a high autoantibody titer, happen simultaneously. This can explain why DHF does not occur during the primary DENV infections, which only have viremia as the first hit but not a high autoantibody titer, or during the convalescence phase, which has a high autoantibody titer as the second hit but not viremia as the first hit.

Clinical studies [38] indicate that the development of DHF coincides with the co-occurrence of viremia and DENV antibody production (Figure 6). Accordingly, our hypothesis is that DHF manifests only when both hits occur simultaneously, which explains why DHF does not occur during the initial infection (which has viremia as the first hit but lacks a high antibody titer as the second hit) or during the convalescence phase (which has a high antibody titer as the second hit but lacks viremia as the first hit). Therefore, a two-hit mouse model capable of inducing dengue-associated hemorrhage pathogenesis was previously presented in our research [6,7]. Due to the possibility of autoantibody fractions being present in DENV-elicited antibodies [4,5,6,7,8,9], secondary infections may generate a higher production of DENV antibodies (Figure 6), and potentially result in clinical symptom progression in DHF, even with comparable viremia levels to initial DENV infections. In accordance with the disease progression of DHF, viremia (virion-associated EIII) and DENV-elicited antibodies are considered to be the potential first and second hits, respectively. As a result, we injected experimental mice with EIII-SNPs and antiplatelet antibodies sequentially.

In fact, the local Shwartzman reaction, known as a phenomenon triggered by LPS challenges, involves a two-step process that has been reported to induce hemorrhage pathogenesis [39]. Initially, a low dose of LPS is administered, which activates the immune system and leads to the release of pro-inflammatory mediators. This initial step sensitizes the tissue for the subsequent impact. In the second step, a higher dose of LPS is introduced, resulting in a robust inflammatory response characterized by tissue damage, vascular leakage, the formation of thrombi, and mortality [39]. It is important to note that a single injection of either LPS dosage does not lead to such pathogenesis. These findings highlight the potential of the two-hit model, utilizing pathogen (LPS)-induced hemorrhagic manifestations, as an effective approach for studying the pathogenesis resulting from multiple viral factor challenges in immune competent mice. Notably, some of these symptoms resemble those observed in DHF. In our experimental study, we administered sequential injections of LPS and LPS or EIII-SNPs and antiplatelet antibodies to mice, which indeed induced comparable thrombocytopenia, hemorrhage, and the release of pro-inflammatory cytokines (Figures S2–S4).

While the two-hit dengue mouse model may not fully replicate the full complexity of the human disease, it offers several advantages over various single-hit models. Firstly, unlike some models that rely on immunocompromised animals to induce severe disease [40], the two-hit model enables challenges of viral factors to elicit DHF-like pathogenesis in immune-competent mice. Secondly, the two-hit model requires DENV virion levels equivalent to DHF to induce DHF-like pathogenesis and mortality in immune-competent mice, whereas single-hit models typically require significantly higher levels of DENV virions. Thirdly, single-hit models usually induce only a single potentiation of pro-inflammatory cytokines, while the two-hit model elicits multiple disease markers recognized by the World Health Organization (WHO) for DHF [41,42], including thrombocytopenia, cytokine storm, liver damage, vascular leakage, and lethality. Lastly, the two-hit model follows a more natural course (Figure 6); better explaining the paradoxical phenomenon of increased risk of lethal DHF during secondary DENV infections. Thus, the two-hit model proves to be a valuable tool.

Despite these successes, the two-hit dengue mouse model has its limitations. Firstly, the specific candidates for the first and second hits have not been clearly identified yet. For example, both EIII and NS1 have been shown to exhibit cytotoxicity and are produced in large quantities during DHF, making them potential candidates for the first hit [7,26,27,43]. Secondly, it is known that DENV proteins elicit a wide range of autoantibodies, all of which could be considered potential second hits [5,8,9,25]. Lastly, due to the lack of clarity regarding the first and second hit candidates, the potential combinations of these hits become numerous, hindering further characterization. This is why in this study, in addition to characterization of the first-hit EIII, we aim to address the pathogenesis associated with antiplatelet antibodies in the two-hit model by utilizing anti-CD41 Ig, to specify the antiplatelet autoantibody fraction is sufficient to serve as a second hit in the two-hit mouse model.

Injection of antiplatelet Ig alone in mice typically causes thrombocytopenia [44] but does not result in hemorrhage [6]. This suggests that antiplatelet Ig alone may not be sufficient to induce vascular damage. Given the high cytotoxicity of EIII-SNPs towards endothelial cells (Figure 1), it is reasonable to hypothesize that sequential administration of EIII-SNPs and antiplatelet Ig could lead to intensified thrombocytopenia and hemorrhage. Therefore, we may consider agents like EIII, EIII-SNPs, and EIII-containing DENV virions as potential triggers of vascular damage (the first hit), while antiplatelet Ig acts as a secondary factor, exacerbating platelet dysfunction, and increasing the risk of bleeding (the second hit). The detailed mechanism underlying the pathogenesis induced by the dengue two-hit mouse model, as well as its ability to replicate DHF pathogenesis, warrants further investigation.

In this report, our data suggest that EIII-SNPs have advantages over the soluble EIII to perform a two-hit dengue hemorrhage mouse model because they induce enhanced cytotoxicity against endothelial cells and display higher cytotoxicity when compared to the GST-SNPs and respective treatments of EIII and SNPs alone. Additionally, EIII-SNPs induce exacerbated thrombocytopenia, worsen hemorrhage pathogenesis, and the induction of pro-inflammatory cytokines TNF-α, IL-1β, IL-6, and anti-inflammatory cytokine IL-10 in mice. Moreover, EIII-SNPs induce an exacerbated suppression of anti-coagulant activated protein C levels in mice, indicating that EIII-SNP may be a more potent material for the development of dengue two-hit hemorrhage pathogenesis mouse models than soluble EIII. The enhancements made to this model could potentially pave the way for further progress in the development of therapeutic strategies for severe dengue infections.

## 4. Materials and Methods

### 4.1. Recombinant Proteins

DENV-2 strain PL046 (GenBank accession number AJ968413.1) was used in this study. The DENV-EIII DNA containing plasmid (DENV-2EIII/pET21b; amino acids 578–674) was used to produce EIII recombinant protein [7,26,27]. The soluble recombinant proteins glutathione-S transferase (GST) and EIII were obtained from cultured *Escherichia coli* bacteria, following induction with isopropyl β-D-1-thiogalactopyranoside, and were subsequently purified using previously described methods [6,7,45]. To minimize the presence of endotoxin (lipopolysaccharide, LPS; <1 EU/mg protein) contamination, the lysate and resin-packed column were washed with a buffer containing 8 M urea, 100 mM NaH_2_PO_4_, 10 mM Tris-HCl (pH = 6.3), and 1% Triton X-114. The EIII was eluted with a buffer of 8 M urea, 100 mM NaH_2_PO_4_, 10 mM Tris-HCl (pH = 4.5), and 300 mM imidazole, and then refolded using a linear 4-0 M urea gradient in a dialysis buffer containing 2 mM reduced glutathione, 0.2 mM oxidized glutathione, 80 mM glycine, 1 mM EDTA, 50 mM Tris-HCl, 50 mM NaCl, and 0.1 mM phenylmethylsulfonyl fluoride for 2–3 h at 4 °C. The EIII protein’s purity was around 90%, and only batches with LPS contamination levels below 1 EU/mg of protein were utilized. Furthermore, it was preserved in −80 °C freezers in 50% glycerol and 50% PBS buffer until use. A Limulus Amoebocyte Lysate QCL-1000 kit (Lonza, Walkersville, MD, USA) was utilized to keep track of the LPS contamination [45,46,47]. The same batch of EIII as previously reported was used in this study [7,26,27].

### 4.2. Experimental Mice

Mice of the C57BL/6J strain that were of the wild-type (WT) and aged between 8 to 12 weeks were procured from the National Laboratory Animal Center in Taipei, Taiwan [13,14,25]. These experimental mice were housed in a specific pathogen-free environment in the Animal Center of Tzu-Chi University, where lighting and temperature were controlled, and they were given unrestricted access to filtered water and food. Around 100 wild-type mice were employed for the study. The Animal Care and Use Committee of Tzu-Chi University, Hualien, Taiwan, approved all the experimental procedures for investigating these animals, with an approval ID of 110024.

### 4.3. Preparation of Protein Coated SNPs

Recombinant protein GST and EIII-coated SNPs (GST-SNPs and EIII-SNPs) were prepared right before the experiments. To prepare GST-SNPs and EIII-SNPs, recombinant GST and EIII (1 mg/mL) were incubated with silica beads (50 nm, 1 mg/mL, Merck/MilliporeSigma, Burlington, MA, USA) [48] in PBS for 1 h at 37 °C. After blocking with 5% bovine serum albumin for additional 30 min and PBS washes, GST-SNPs and EIII-SNPs were ready to use. The detection of EIII coating on the SNPs was accomplished by measuring the streptavidin-phycoerhthrin-Cy5 binding signal to biotinylated EIII on the SNPs using flow cytometry (Figure S1).

### 4.4. Measurements of Endothelial Cell Survival and Injury

The survival rate of human endothelial HMEC-1 cells [7] was assessed post-treatment with various substances, including vehicle, GST, EIII (0.6 μM, a dose of EIII functionally equivalent to the viral-load range of circulating DENV viron-associated EIII in DHF patients, determined by activated partial thromboplastin clotting time assay [26]), and GST-SNPs and EIII-SNPs (with a similar dose of soluble protein). The WST-1 kit (Roche Life Science, Penzberg, Germany) was employed to determine cell viability following the manufacturer’s instructions [8,49,50]. The succinate-tetrazolium reductase system is responsible for breaking down the tetrazolium salt WST-1 into a soluble formazan. This system functions only in metabolically active cells, and the resulting formazan can be measured at 450 nm using an ELISA reader (Molecular Devices, Sunnyvale, CA, USA) [51]. To measure EIII and EIII-SNP-induced endothelial cell injury, a soluble thrombomodulin ELISA Kit (Abcam, Cambridge, UK) was used as described [7].

### 4.5. Two-Hit Challenges with EIII and EIII-SNPs along with Antiplatelet Immunoglobulin (Ig) to Induce a Local Shwartzman Reaction-like Response and the Cytokine and Anticoagulant Analyses

To induce local Shwartzman reaction-mediated hemorrhage in mice using traditional methods, two sequential subcutaneous LPS injections are required over a period of two consecutive days [39]. In our mouse model involving two hits, the mice were initially given EIII through subcutaneous injection, with a dosage of 2 mg/kg mouse body weight, equivalent to the viral-load range of circulating DENV viron-associated EIII in DHF patients [7,26]. This was followed by a subcutaneous injection of antiplatelet IgGs (0.2 mg/kg), specifically the rat monoclonal MWReg30 (BD Biosciences, San Jose, CA, USA) [7], which is established for ITP induction [44], after 24 h. In similar experiments, we administered an equivalent amount of SNP-coated EIII, as indicated in the EIII experiment. Anesthesia was induced 5 min before each injection (e.g., vehicle, recombinant proteins, protein-SNPs, and Igs) by intraperitoneal injection of 2.5% Avertin solution (in saline, 10 mL/kg). Twenty-four hours after the injection of antiplatelet Ig, we analyzed platelet counts [Analyzer KX-21N; Sysmex [44], degree of hemorrhage, levels of cytokines IL-1β. IL-6, IL-10, and TNF-α (ELISA, e-Bioscience, Thermo Fisher Scientific, Waltham, MA, USA), and the levels of expression of anti-coagulant proteins anthithrombin III (chromogenic, Sekisui Diagnostica, Burlington, MA, USA) and protein C (chromogenic, Sekisui Diagnostica). For platelet count analysis, blood samples were collected and placed into polypropylene tubes containing an anticoagulant solution of acid-citrate-dextrose (38 mM citric acid, 75 mM sodium citrate, and 100 mM dextrose) [49,52]. To obtain platelet-poor plasma for the analysis of cytokines and anticoagulant proteins, the samples were subjected to centrifugation at 1500× *g* for 20 min, followed by a subsequent centrifugation at 15,000× *g* for 3 min to remove contaminant cells from the plasma. Comparisons of platelet counts, hemorrhage score and pro-inflammatory cytokines IL-1 and TNF among different two-hit challenged mouse groups [e.g., LPS + LPS first hit LPS (200 μg/kg) + second hit LPS (1.2 mg/kg), LPS + anti-CD41 Ig, EIII + anti-CD41 Ig, and EIII-SNPs + anti-CD41 Ig] were indicated (Figures S2–S4).

### 4.6. Quantification for the Degree of Hemorrhage Using Digitized Images

While the grading of hemorrhage in the local Shwartzman reaction can be assessed using a subjective arbitrary scale of 0 to 4 [53,54], we aimed to develop a more objective quantification protocol based on established methods [6,7]. Standardized conditions were used to capture images of the hemorrhagic lesions [illumination density of 200 lux, 20 W Phillips fluorescent lamp (Phillips Taiwan, Taipei, Taiwan), Canon IX-US-860IS camera (Canon Taiwan, Taipei, Taiwan), with a sample-to-camera distance of 7 cm]. The digitized images (RGB mode, 0.75 × 0.6 cm^2^, 600 dpi) were then analyzed using Photoshop software (v7, Adobe) to obtain red and green signals without any brightness or contrast adjustments. To further measure the red and green intensities in a particular image of the hemorrhagic lesion, we utilized Image J software (v1.46, NIH). An approximate severity value for the hemorrhage was calculated by subtracting the image intensity of the red images from that of the green images, as previously described [6,7].

### 4.7. Statistical Analyses

The quantifiable data were analyzed by calculating the means, standard deviations, and statistics using Microsoft Office Excel 2003, SigmaPlot 10, and SPSS 17. To test the data’s significance, a 1-way ANOVA was conducted, followed by the post hoc Bonferroni-corrected *t*-test. A threshold of statistical significance was established with a probability of type 1 error α = 0.05.

## Data Availability

The datasets used and analyzed during the current study are available from the corresponding author on reasonable request.

## Supplementary Materials

The following supporting information can be downloaded at: https://www.mdpi.com/article/10.3390/ijms24119270/s1.
