# Supplementary material for: Simulation of Hemorrhage Pathogenesis in Mice through Dual Stimulation with Dengue Envelope Protein Domain III-Coated Nanoparticles and Antiplatelet Antibody
Source: Int J Mol Sci. 2023 May 25;24(11):9270. doi: 10.3390/ijms24119270 (PMC10253347; doi:10.3390/ijms24119270)
Supplement: Supplementary file 1 [file ijms-24-09270-s001.zip › ijms-2387073-supplementary.pdf]

Supplementary information

Simulation of hemorrhage pathogenesis in mice through dual stimulation with dengue envelope protein domain III-coated nanoparticles and antiplatelet antibody

Te-Sheng Lien [1], Der-Shan Sun [1], Wen-Sheng Wu [2], and Hsin-Hou Chang [1*]

[1] Department of Molecular Biology and Human Genetics, Tzu-Chi University, Hualien 970, Taiwan; alan211@mail.tcu.edu.tw; dssun@mail.tcu.edu.tw; hhchang@mail.tcu.edu.tw

[2] Division of General Surgery, Department of Surgery, Hualien Tzu Chi Hospital, Buddhist Tzu Chi Medical Foundation, Hualien 970, Taiwan; wuws@gms.tcu.edu.tw

* Correspondence: hhchang@mail.tcu.edu.tw; Tel.: 886-3-8565301 ext 2667

Key words: dengue virus; dengue envelope protein domain III; dengue hemorrhage fever; hemorrhage; anti-platelet antibody; silica nanoparticles; inflammation; cytokines; anti-coagulants; two-hit model

* To whom correspondence and reprint requests should be addressed.

Hsin-Hou Chang Ph.D. Room D407, Tzu-Chi University, No. 701, Section 3, Chung-Yang Road, Hualien 97004, Taiwan. Tel: 886-3-8565301 ext 2667. Fax: 886-3-8578386. E-mail: hhchang@mail.tcu.edu.tw

Figures S1-S4: pages 2-5

Figure S1

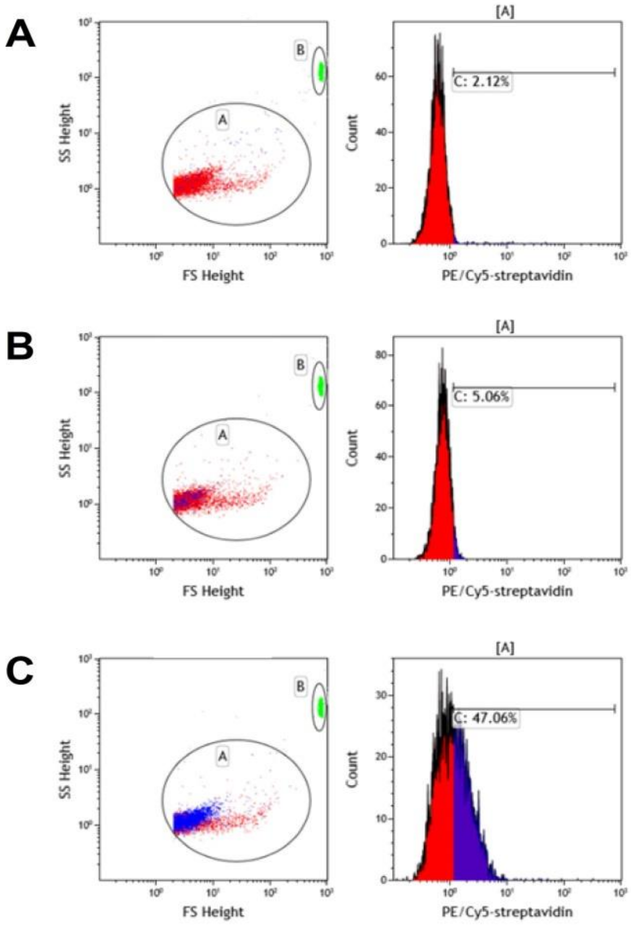

Figure S1. The detection of EIII coating on the SNPs was accomplished by measuring the streptavidin-phycoerhthrin-Cy5 (PE/Cy5) binding signal to biotinylated EIII on the SNPs using flow cytometry. Examples of flow cytometry gating graphs of (A) SNPs only (without staining) groups, (B) SNPs + streptavidin-PE/Cy5 groups, and (C) EIII-SNPs + streptavidin-PE/Cy5groups.

Figure S2

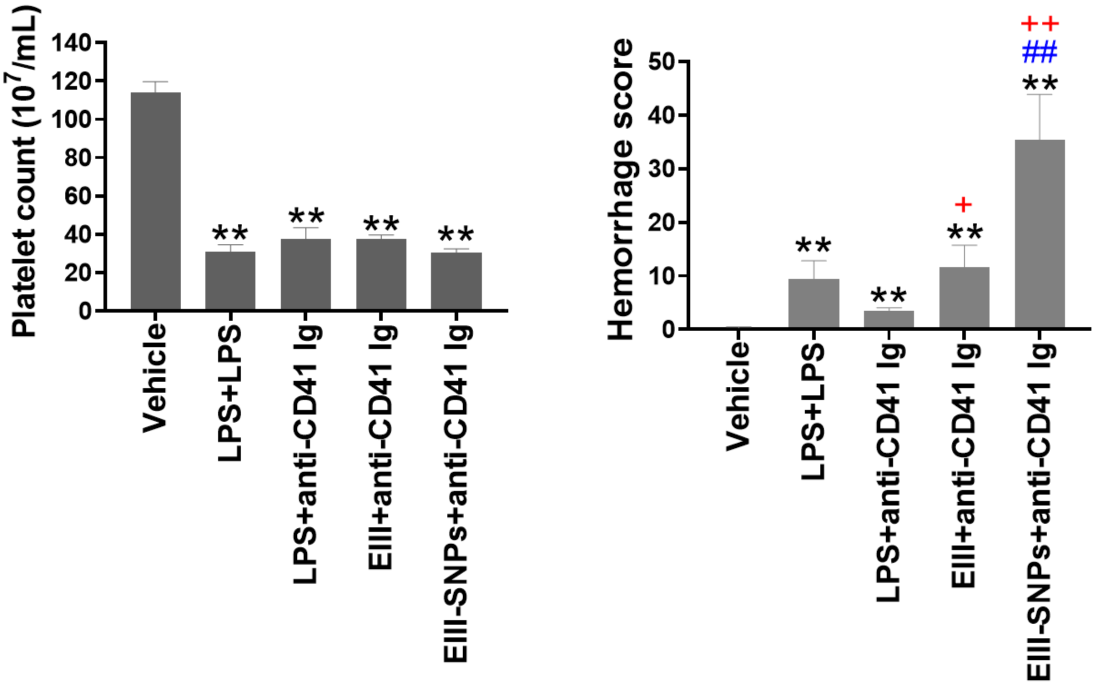

Figure S2. Comparisons of platelet counts and hemorrhage score after different two-hit treatments in mice. Platelet counts and hemorrhage score were analyzed in mice groups without (vehicle), and with different 2-hit treatments [LPS (200 μg/kg) + LPS (1.2 mg/kg), LPS (200 μg/kg) + anti-CD41 Ig, EIII + anti-CD41 Ig, and EIII-SNPs + anti-CD41 Ig]. N = 6. ** $P < 0.01$, vs. vehicle control groups; ## $P < 0.01$, vs. respective LPS + LPS groups; + $P < 0.05$, ++ $P < 0.01$, vs. respective LPS + anti-CD41 Ig groups. Data are presented as mean ± SD.

Figure S3

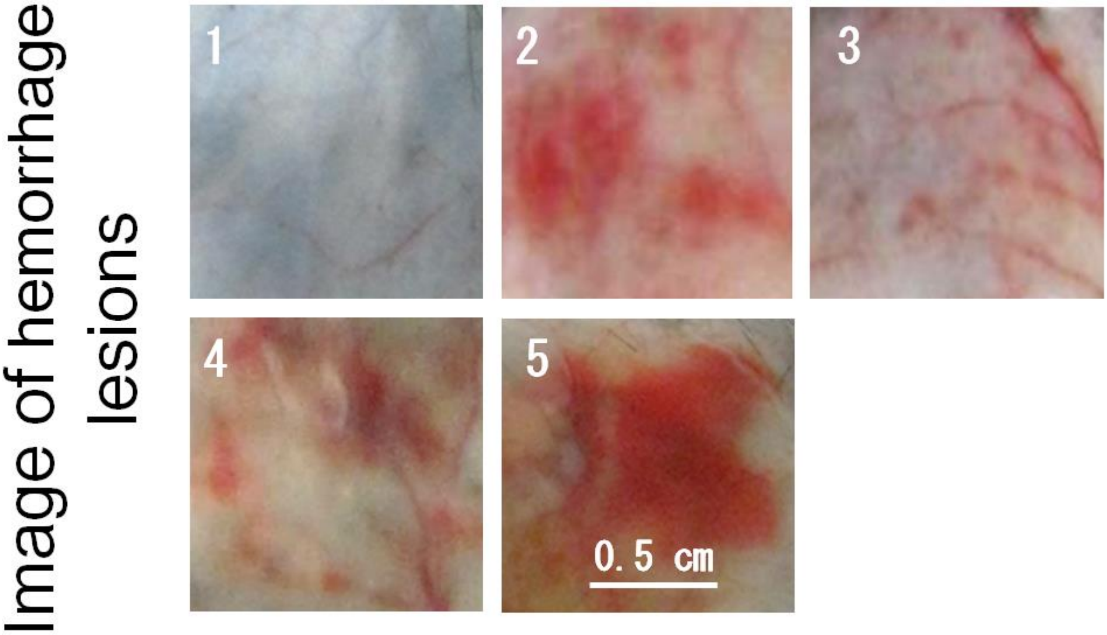

Figure S3. Images of hemorrhage lesions of mice after different two-hit treatments. Example images of hemorrhage lesions that were used for calculating the hemorrhage score (Figure S2) in mice. (1) vehicle, (2) LPS + LPS, (3) LPS + anti-CD41 Ig, (4) EIII + anti-CD41 Ig, (5) EIII-SNPs + anti-CD41 Ig. Scale bar: 0.5 cm.

Figure S4

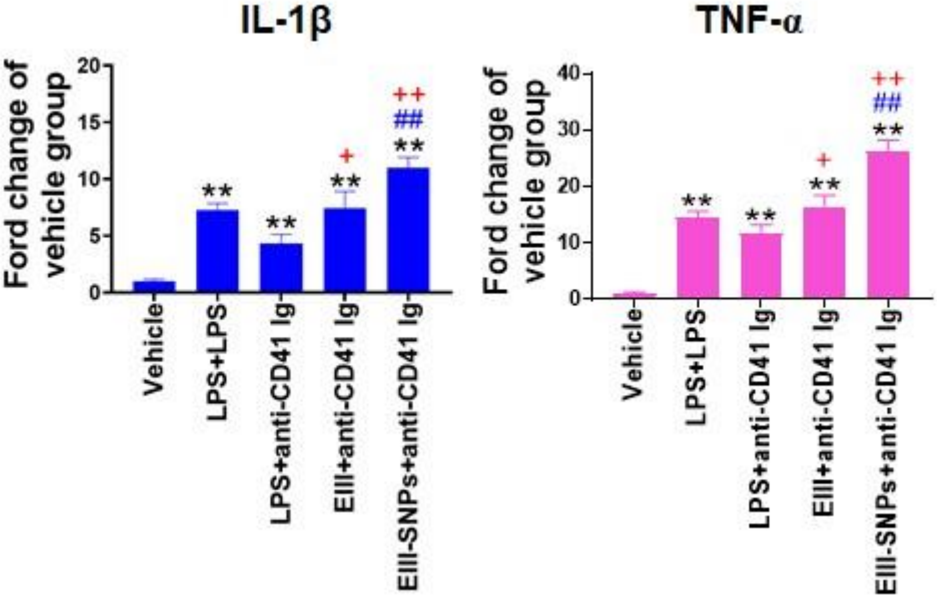

Figure S4. Comparisons of circulating cytokine IL-1β and TNF-α levels after different two-hit treatments in mice. Enzyme-linked immunosorbent assay was performed to examine the expression of proinflammatory cytokines IL–1β, and TNF–α in mice groups without (vehicle), and with different 2-hit treatments [LPS (200 μg/kg) + LPS (1.2 mg/kg), LPS (200 μg/kg) + anti-CD41 Ig, EIII + anti-CD41 Ig, and EIII-SNPs + anti-CD41 Ig]. N = 6. ** $P < 0.01$, vs. vehicle control groups; ## $P < 0.01$, vs. respective LPS + LPS groups; + $P < 0.05$, ++ $P < 0.01$, vs. respective LPS + anti-CD41 Ig groups. TNF–α, tumor necrosis factor–α; IL–1β, interleukin–1β. Data are presented as mean ± SD.
